# Sudden Death of a Toddler With Rubinstein-Taybi Syndrome: Forensic Investigation and Pathophysiological Correlation

**DOI:** 10.7759/cureus.111984

**Published:** 2026-07-03

**Authors:** Ioannis Ketsekioulafis, Athina Tousia, Konstantinos Katsos, Artemis A Dona, Dimitrios Vlachodimitropoulos, Chara Spiliopoulou, Emmanouil I Sakelliadis

**Affiliations:** 1 Department of Forensic Medicine and Toxicology, National and Kapodistrian University of Athens School of Medicine, Athens, GRC

**Keywords:** immunodeficiency, pediatric autopsy, respiratory infection, rubinstein-taybi syndrome, sudden pediatric death

## Abstract

Sudden death in childhood is a particularly demanding subject of forensic investigation, especially when a known genetic syndrome coexists. We present the case of the sudden death of a three-year-old female toddler diagnosed with Rubinstein-Taybi syndrome, who was found pulseless and apneic at home and pronounced dead shortly after being transferred to a children's hospital. A comprehensive forensic investigation was conducted, including an autopsy, histological examination, and toxicological analysis. The histopathological findings revealed extensive lesions of severe bronchopneumonia with necrosis, abscess formation, and interstitial pneumonitis, without evidence of trauma or toxic etiology. The cause of death was attributed to severe respiratory infection in a child with Rubinstein-Taybi syndrome. Although immunological abnormalities have been described in some patients with Rubinstein-Taybi syndrome, no direct immunological evaluation was available in the present case. The case highlights the importance of individualized evaluation of pediatric deaths and the correlation of clinical, genetic, and pathologic data.

## Introduction

Rubinstein-Taybi syndrome (RTS) is a rare congenital syndrome characterized by highly dysmorphic craniofacial features, broad thumbs and big toes, postnatal growth retardation, and varying degrees of intellectual disability. Its estimated incidence ranges from 1:100,000 to 1:125,000 live births, with most cases occurring sporadically. At the molecular level, the syndrome is primarily attributable to heterozygous pathogenic mutations or microdeletions in the CREBBP gene and, less commonly, in EP300, which encode transcriptional co-activators with histone acetyltransferase activity that are critical for gene expression regulation and chromatin remodeling [1-9].

In addition to the classic phenotypic manifestations, RTS is now recognized as a multisystem disease with significant clinical heterogeneity. Patients often present with congenital heart disease, urinary tract abnormalities, ophthalmological abnormalities, feeding difficulties, and skeletal abnormalities. Growth retardation is a common finding from infancy and may be exacerbated by recurrent infections, nutritional problems, and coexisting metabolic or endocrine disorders [2,4,10,11].

In recent years, increasing interest has been given to the immune dysfunction observed in patients with RTS. Multiple reports have described an increased incidence of recurrent and severe infections in some patients with RTS, raising the possibility of underlying immune abnormalities. Both humoral and cellular immune disorders have been described, including hypogammaglobulinemia, inadequate immune response to vaccines, abnormalities in lymphocyte populations, and evidence of functional "exhaustion" of the immune system. Several mechanisms have been proposed to explain these observations, including impaired immune cell maturation, altered lymphocyte function, chronic immune activation, and dysregulated immune responses; however, the spectrum and clinical significance of these findings remain incompletely understood [12-15].

Despite the above, the role of the immune system in RTS is frequently underestimated in clinical practice and is not systematically incorporated into patient diagnosis and follow-up. As a result, immunological complications may be diagnosed late, with increased morbidity and a negative impact on physical growth, neurodevelopmental course, and overall prognosis.

Sudden death in children with RTS is a rare but clinically and forensically critical event, requiring detailed investigation and careful interpretation of the findings. Although recurrent respiratory infections have been described in patients with Rubinstein-Taybi syndrome, fatal respiratory infection-related sudden death remains rarely reported in the literature. Consequently, such cases represent a significant diagnostic and forensic challenge, requiring careful integration of clinical history, genetic information, autopsy findings, and histopathological examination.

## Case presentation

A three-year-old female toddler was found unconscious in her home by her mother during an attempt to wake her up for her transfer to kindergarten. Her mother and her grandparents immediately transported her to the local Children's General Hospital, where she was found to be pulseless, apneic, with central cyanosis and mydriasis. Α SARS-CoV-2 test was performed, which was negative. Despite the application of basic cardiopulmonary resuscitation measures, death was determined two hours after hospital admission. 

Regarding family history, the mother was 18 years old and the father 20 at the time of the child's birth. The parents were unrelated and unmarried. The child lived with her mother and grandparents. According to the available information, both parents are reported as drug users. No known family history of genetic diseases, congenital anomalies, or sudden childhood deaths was reported.

The perinatal history revealed that the mother had multiple episodes of urinary tract infections during pregnancy, as well as iron deficiency anemia, which necessitated the administration of blood transfusions. The placenta was characterized as prematurely aged. During prenatal ultrasound examination, intrauterine growth retardation (IUGR) was detected, while pathological findings were also recorded in the non-stress test (NST). Due to the above, it was decided to perform a cesarean section at 36 weeks and four days of gestation.

After delivery, dysmorphic facial and limb features were observed, along with delayed physical and neurodevelopmental milestones. During her subsequent course, the child developed recurrent infections, mainly of the respiratory system, as well as persistent hematological abnormalities, including documented leukocytosis and lymphocytosis according to the available hospital records, which prompted further clinical investigation. In the context of investigating the above findings, whole-exome sequencing (WES) was performed, leading to the diagnosis of Rubinstein-Taybi syndrome.

**Figure 1:**
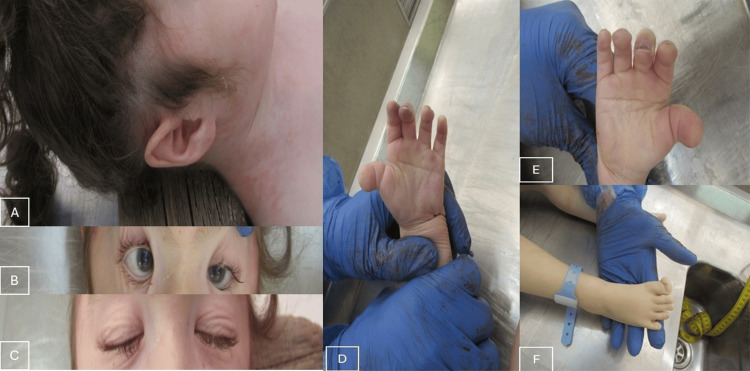
Dysmorphic features consistent with Rubinstein-Taybi syndrome A) Stahl's ears; B) Esotropia of both eyes; C) Downslanted palpebral fissures; D, E) Broad radially deviated thumbs; F) Broad partially duplicated halluces

At autopsy, characteristic deformities consistent with Rubinstein-Taybi syndrome were found, such as Stahl's ears, esotropia of both eyes, downslanted palpebral fissures (which describes the upward-sloping angle of the eye openings where the outer corners are lower than the inner corners), prominent beaked nose, high-arched palate, broad radially deviated thumbs, broad partially duplicated halluces, as well as failure to thrive according to WHO growth charts for age and sex (Figure 1) [16].

As for the postmortem changes, livor mortis was present on the dorsal surface of the body. Evidence of medical manipulations was identified, consistent with an attempt of cardiopulmonary resuscitation, as well as multiple needle-puncture marks on the upper and lower limbs. No recent external injuries were found. 

After the dissection of the body, no injuries were found to the head, thorax, or intra-abdominal organs. Bilateral pleural effusions were observed in the chest. The lungs were markedly congested, with a macroscopically visible ​​reddish-brown area in the middle lobe of the right lung. The heart showed thickening of the left ventricular wall and interventricular septum, without congenital or coronary lesions. Serous fluid was found in the abdominal cavity. The other organs showed no significant macroscopic lesions.

**Figure 2:**
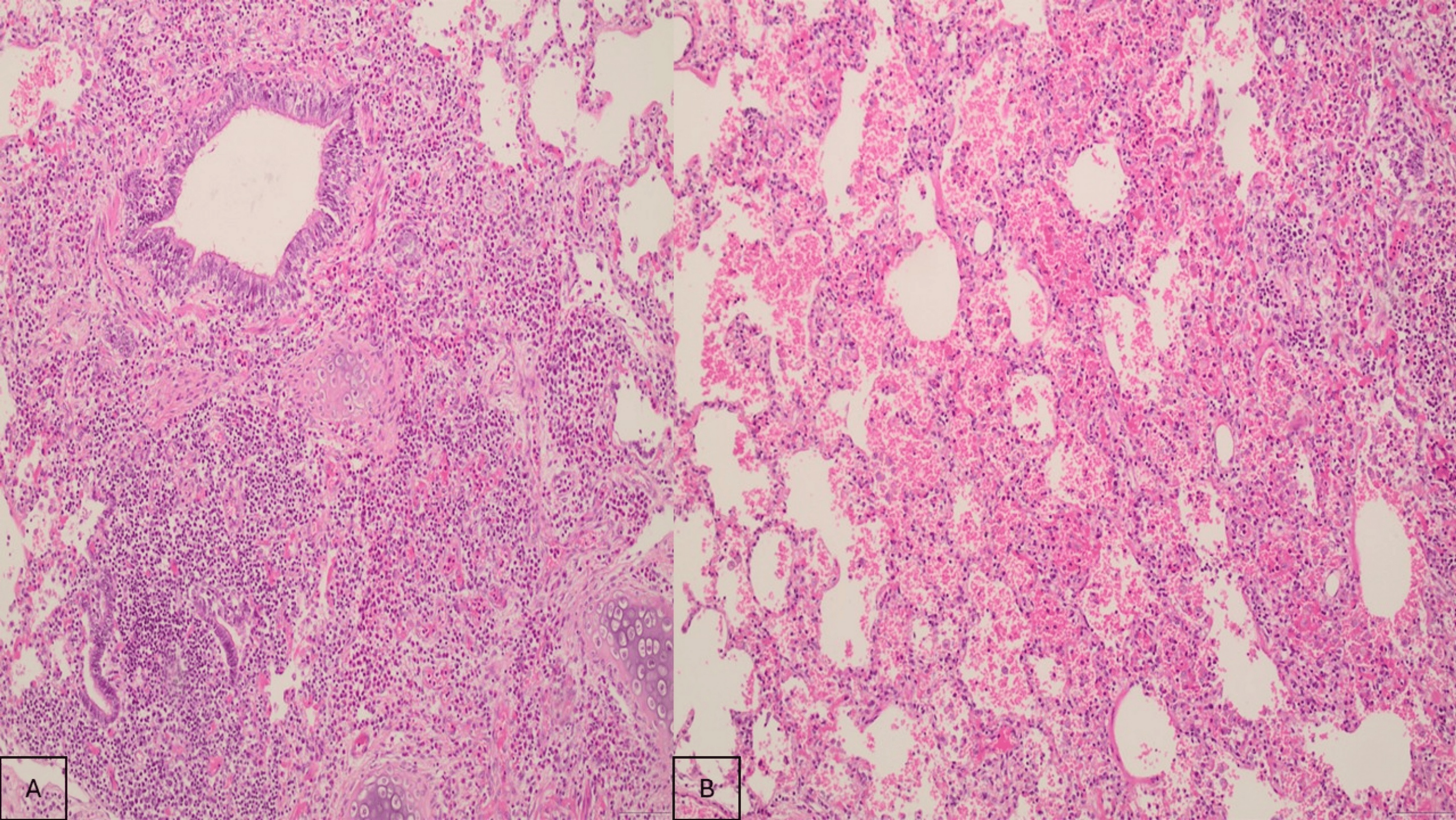
H&E lung specimen (x200 magnification) The lungs appear markedly congested, with severe interstitial pneumonia, focal inflammatory infiltration of the wall of the bronchioles (A), and pulmonary hemorrhage (B).

Histologically, the lungs showed severe bilateral interstitial pneumonitis (Figure 2). 

**Figure 3:**
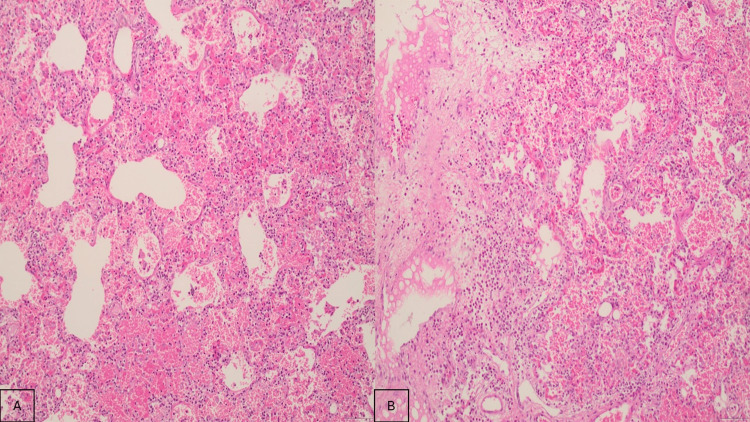
H&E lung specimen (x200 magnification) In the right lung, histopathological examination demonstrated severe bronchopneumonic changes, particularly in the macroscopically reddish-brown region, characterized by extensive areas of necrosis (A) and abscess formation (B).

Extensive lesions of severe bronchopneumonia with areas of necrosis, abscess formation, and pulmonary hemorrhage were identified in the right lung (Figure 3). 

The heart showed mild interstitial edema and ischemic-type lesions (compatible with agonal ischemic changes occurring shortly before death), without disturbance of myocardial architecture. The brain and other organs showed no specific histopathological lesions.

Toxicological examination detected lidocaine at therapeutic concentrations in the blood and urine, a finding compatible with its administration in the context of cardiopulmonary resuscitation. No other toxic or pharmaceutical substances were detected.

Following completion of the forensic investigation, the cause of death was determined to be fatal necrotizing bronchopneumonia with bilateral interstitial pneumonitis. The death was classified as natural and occurred in the setting of Rubinstein-Taybi syndrome.

## Discussion

Sudden death of children is one of the most demanding categories of forensic cases, as it combines intense social and legal interest with the need for cautious medical interpretation. This difficulty is multiplied when the child suffers from a rare genetic syndrome, such as Rubinstein-Taybi syndrome (RTS), whose phenotype includes a multitude of systemic disorders, often with atypical or subclinical manifestations.

In the presented case, the cause of death was attributed to a severe respiratory infection, specifically extensive bronchopneumonia with necrosis, abscess formation, and accompanying interstitial pneumonitis. Although respiratory infections are a common finding in childhood, their fatal outcome in an apparently stable child may raise reasonable questions. The interpretation of death, however, becomes clear when examined in the context of the underlying syndrome.

Rubinstein-Taybi syndrome is characterized by a broad spectrum of clinical manifestations, with an almost universal presence of developmental delay and a high frequency of dysmorphic features, ophthalmological disorders, skeletal abnormalities, and growth disorders [2-11,17]. Of particular importance for the present case are the respiratory features of the syndrome, which include recurrent infections, aspiration, and increased vulnerability of the lower respiratory system. According to the international literature, respiratory infections are one of the most common causes of morbidity in patients with RTS, and are also reported as a factor in increased mortality [2,3,5-7,9-11].

A critical role in understanding the sudden deterioration and death is played by the immunological disorders that accompany the syndrome. Immunological abnormalities, including humoral and cellular immune defects, have been reported in a subset of patients with RTS. Pathophysiologically, immunodeficiency may arise through multiple mechanisms that act in concert. These include the presence of clonal, immature, or functionally deficient immune cells; bone marrow congestion, with subsequent reduction in normal hematopoiesis; and the secretion of inhibitory cytokines. In addition, hypogammaglobulinemia and functional immune depletion due to chronic activation, as observed in lymphoproliferative conditions, have been described [1,12-15,18,19]. However, no immunological investigations were available in the present case; therefore, immune dysfunction cannot be directly demonstrated. Consequently, any contribution of immune abnormalities to the development or severity of the respiratory infection should be regarded as a possible rather than a proven mechanism.

The histopathological findings in the child's lungs confirm the existence of a severe and prolonged inflammatory process, with extensive areas of necrosis and abscess formation, evidence that is consistent with a prolonged infectious process. Although impaired host defense mechanisms may represent one possible explanation, direct evidence supporting immune dysfunction was unavailable in the present case. 

Cardiac findings were also carefully evaluated during the forensic investigation. Gross examination demonstrated thickening of the left ventricular wall and interventricular septum, while histological examination revealed mild interstitial edema, minimal focal inflammatory infiltration, and ischemic-type myocardial changes. No evidence of myocarditis, significant myocardial inflammation, myocardial architectural disorganization, coronary artery abnormalities, or other structural cardiac lesions capable of independently explaining death was identified. The observed ischemic-type alterations were considered compatible with terminal myocardial ischemic injury occurring shortly before death rather than a primary cardiac disease process. In contrast, the lungs demonstrated extensive necrotizing bronchopneumonia, abscess formation, pulmonary hemorrhage, and bilateral interstitial pneumonitis, providing a clear pathological substrate for the fatal outcome. Nevertheless, as in all cases of sudden pediatric death, occult functional cardiac abnormalities cannot be completely excluded.

Alternative and contributory mechanisms were also considered during the forensic evaluation. Rubinstein-Taybi syndrome has been associated with craniofacial abnormalities, feeding difficulties, recurrent respiratory infections, and sleep-disordered breathing, all of which may increase vulnerability to severe pulmonary disease. Aspiration-related respiratory compromise, therefore, represents a potential contributory factor in some patients with RTS. Additionally, although sepsis could not be confirmed in the absence of premortem laboratory and microbiological investigations, the extensive pulmonary pathology raises the possibility of systemic inflammatory involvement. Occult functional cardiac abnormalities, metabolic disturbances, or other non-structural mechanisms cannot be completely excluded. A further limitation of the present case is the absence of detailed clinical information regarding symptoms immediately preceding death, such as fever, cough, respiratory distress, or other manifestations of acute infection. Furthermore, microbiological investigations, immunological studies, and other ancillary laboratory evaluations were unavailable, limiting the ability to further characterize the infectious process and potential patient-related predisposing factors. Nevertheless, the severity and extent of the pulmonary lesions identified at autopsy and histological examination provided the most convincing pathological substrate for the fatal outcome.

From a forensic perspective, the incident did not reveal any evidence of trauma, abuse, or toxic etiology. The detection of lidocaine at therapeutic concentrations was documented to be due to its administration in the context of cardiopulmonary resuscitation and is not causally related to death. The systematic correlation of clinical, macroscopic, histological, and toxicological findings allowed the safe exclusion of exogenous factors.

Finally, the immediate cause of death was fatal necrotizing bronchopneumonia with bilateral interstitial pneumonitis. Rubinstein-Taybi syndrome represented the underlying disease, while recurrent infections, growth failure, and other syndrome-related vulnerabilities may have contributed to the severity of the infectious process. The mechanism of death was most likely respiratory failure secondary to extensive pulmonary disease. Based on the clinical history, autopsy findings, histopathological examination, and toxicological analysis, the manner of death was classified as natural. The present case clearly highlights that children with rare genetic syndromes constitute a special category of patients, in whom even seemingly common pathological conditions may have a disproportionately severe outcome. For the forensic pathologist, knowledge of the pathophysiology of such syndromes and understanding of the specificities of pediatric morbidity are crucial for the correct interpretation of the cause of death and the avoidance of erroneous conclusions.

## Conclusions

This case highlights the importance of comprehensive forensic investigation in sudden pediatric deaths occurring in children with rare genetic syndromes. The autopsy and histopathological findings demonstrated fatal necrotizing bronchopneumonia with interstitial pneumonitis in a child with Rubinstein-Taybi syndrome. Although immunological abnormalities have been reported in association with RTS, their contribution to the present case could not be directly evaluated because immunological and microbiological investigations were unavailable. Nevertheless, the case underscores the value of integrating clinical history, genetic information, autopsy findings, and histopathological examination in establishing the cause of death and improving our understanding of rare disease-associated mortality.
